# Could Insulin Be a Better Regulator of Appetite/Satiety Balance and Body Weight Maintenance in Response to Glucose Exposure Compared to Sucrose Substitutes? Unraveling Current Knowledge and Searching for More Appropriate Choices

**DOI:** 10.3390/medsci12020029

**Published:** 2024-06-06

**Authors:** Georgios Antasouras, Antonios Dakanalis, Maria Chrysafi, Sousana K. Papadopoulou, Ioulia Trifonidi, Maria Spanoudaki, Olga Alexatou, Agathi Pritsa, Aikaterini Louka, Constantinos Giaginis

**Affiliations:** 1 Department of Food Science and Nutrition, School of Environment, University of Aegean, 81400 Lemnos, Greece; g.antasouras@gmail.com (G.A.); fnsm22030@fns.aegean.gr (M.C.); rd.olga.alexatou@gmail.com (O.A.); loukathy612@gmail.com (A.L.); 2 Department of Mental Health, Fondazione IRCCS San Gerardo dei Tintori, Via G.B. Pergolesi 33, 20900 Monza, Italy; antonios.dakanalis@unimib.it; 3 Department of Medicine and Surgery, University of Milan Bicocca, Via Cadore 38, 20900 Monza, Italy; 4 Department of Nutritional Sciences and Dietetics, School of Health Sciences, International Hellenic University, 57400 Thessaloniki, Greece; souzpapa@gmail.com (S.K.P.); maryspan1@gmail.com (M.S.); agpritsa@ihu.gr (A.P.); 5 Department of Clinical Biochemistry, KAT General Hospital, 14561 Athens, Greece; trifoula@yahoo.gr

**Keywords:** acesulfame, anorexigenic peptides, aspartame, body weight, hypothalamus, insulin, non-nutritive sweeteners, orexigenic peptides, saccharin, stevia

## Abstract

Background: Insulin exerts a crucial impact on glucose control, cellular growing, function, and metabolism. It is partially modulated by nutrients, especially as a response to the intake of foods, including carbohydrates. Moreover, insulin can exert an anorexigenic effect when inserted into the hypothalamus of the brain, in which a complex network of an appetite/hunger control system occurs. The current literature review aims at thoroughly summarizing and scrutinizing whether insulin release in response to glucose exposure may be a better choice to control body weight gain and related diseases compared to the use of sucrose substitutes (SSs) in combination with a long-term, well-balanced diet. Methods: This is a comprehensive literature review, which was performed through searching in-depth for the most accurate scientific databases and applying effective and relevant keywords. Results: The insulin action can be inserted into the hypothalamic orexigenic/anorexigenic complex system, activating several anorexigenic peptides, increasing the hedonic aspect of food intake, and effectively controlling the human body weight. In contrast, SSs appear not to affect the orexigenic/anorexigenic complex system, resulting in more cases of uncontrolled body weight maintenance while also increasing the risk of developing related diseases. Conclusions: Most evidence, mainly derived from in vitro and in vivo animal studies, has reinforced the insulin anorexigenic action in the hypothalamus of the brain. Simultaneously, most available clinical studies showed that SSs during a well-balanced diet either maintain or even increase body weight, which may indirectly be ascribed to the fact that they cannot cover the hedonic aspect of food intake. However, there is a strong demand for long-term longitudinal surveys to effectively specify the impact of SSs on human metabolic health.

## 1. Introduction

**Figure 1:**
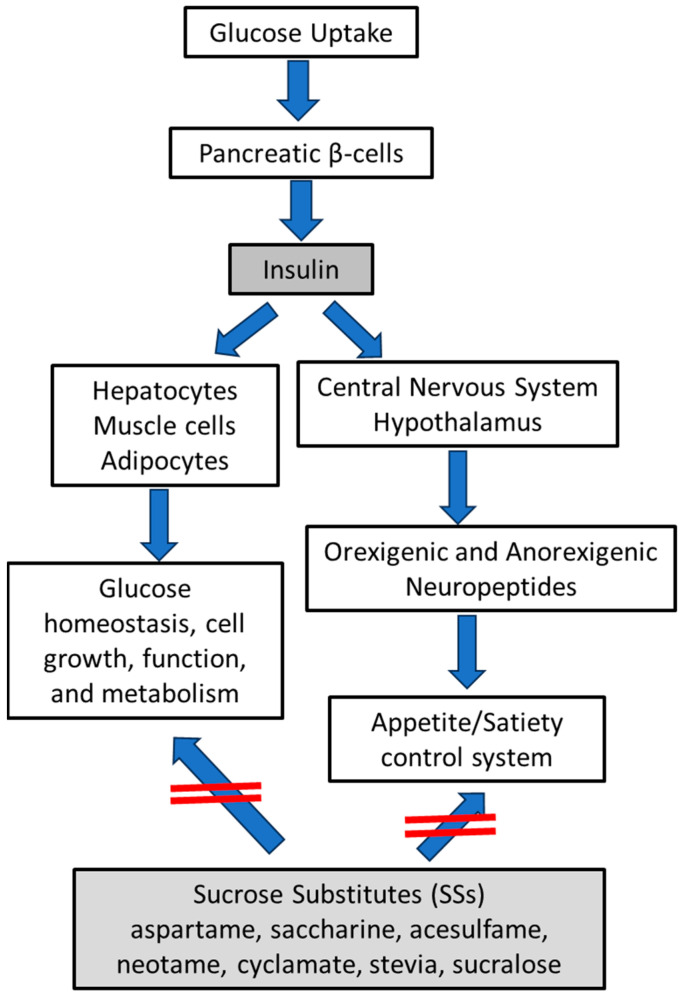
The two potential actions of insulin as regulator of glucose homeostasis and appetite/satiety control system and the role of SSs.

Insulin constitutes a peptide hormone, which exerts a crucial impact on glucose homeostasis, cellular growing, function, and metabolism [1]. The biosynthesis of insulin takes place in the β-cells of the pancreas islets, and insulin transcription and translation are partially modulated by nutrients, especially as a response to increased blood glucose levels during the consumption of foods including carbohydrates [2]. Once insulin is inserted into the bloodstream, it then transfers to the adipocytes, hepatocytes, and muscle cells of the human body. Afterward, insulin molecules bind to the insulin receptors (IRs) of these cells, which are activated to receive the ingested glucose through glucose transporters [3]. Beyond the above insulin action, this hormone can also penetrate the blood–brain barrier (BBB), a great discriminating semi-permeation border of vascular endothelial cells that regulates the transport of solutes, nutrients, and chemicals from the circulatory system to the central nervous system (CNS) [4]. By passing the BBB, insulin can reach diverse regions of the CNS, like the hypothalamus, the amygdala, and the brainstem, in which it exerts diverse utilities by interacting with the broadly expressed IRs [5,6]. The two potential actions of insulin as regulator of glucose homeostasis and appetite/satiety control system and the role of SSs are depicted in Figure 1.

Diverse physiology functions, like energy balance, reproduction, and cognition, are systematically coordinated across the CNS through an interaction between crucial signaling compounds in various interrelated neuronal networks [7]. Specifically, during ingestion procedures, diverse neurochemical events can be activated, promoting signal nutrient availability to the CNS [Gasmi 2022]. Then, the CNS initiates the control of appetite/satiety balance, which is supplementary to nutrient intake or no eating, as well as energy intake/expenditure, which may influence body weight and energy balance [8]. It is currently well-documented that the hypothalamus constitutes the most important brain site in detecting and responding to nutrient alterations, in which neuronal peptides, and especially anorexigenic or orexigenic peptides, are released in accordance with the signaling received from the body periphery, depending on the nutrient’s requirements or deficiencies of the human body [9]. In this aspect, the hypothalamic compartment constitutes the responsible part of the CNS, in which eating behavior is modulated, being highly associated with glucose control levels [10].

There are several hypothalamic neuropeptides or neurohormones that can act by complex networks relating to the main hypothalamic nuclei and contributing to food intake regulation [11]. Both orexigenic and anorexigenic neuropeptides released from the hypothalamus appear to be implicated in the control of appetite/satiety balance and, subsequently, nutrient intake [12]. One of the key signaling molecules in the hypothalamus concerns brain insulin, which can exert a fundamental role in these functions [13]. Notably, brain insulin can exert anorexigenic effects by binding to the IRs of the hypothalamus, in which a complex network of an appetite/hunger control system occurs [14]. Dysregulation of insulin excretion and transportation into the CNS, despite a lack of insulin or resistance to the CNS, has extensively been noted in association with the elderly, obesity, diabetes, and several mental diseases, as well as appetite/satiety imbalances related to eating disorders [15,16].

In the last decades, several sucrose substitutes (SSs) included in foods and beverages have rapidly been developed by the food industry with the aim to minimize excessive calorie intake, which could better control healthy body weight maintenance, reducing the risk of obesity and related diseases [17]. However, SS intake does not influence the appetite/hunger complex system of the hypothalamus, and it cannot satisfy the hedonic aspect of food and beverage intake [18,19]. Thus, it has been supported that they cannot activate the anorexigenic neuropeptides of hypothalamus, enhancing the sense of hunger, and thus the intake of excessive calories on a long-term basis, which may lead to body weigh increase if a well-balance diet is not carefully applied at a daily frequency [18,19]. Although there are a plethora of data from surveys that have explored sweets/beverages, sucrose, and sweetener consumption and body weight, there are only restricted data concerning the association between overall nutritional sweetness and body weight management for a long-term period [20]. Thus, there is not conclusive evidence so far concerning the SSs and their positive or negative effects on human health.

Based on the aforementioned thoughts, the current literature review purposes to critically summarize and scrutinize whether insulin release in response to glucose exposure could be a better choice to control body weight gain and decrease the risk of related diseases compared to the use of SSs in combination with a long-term, and well-balanced diet.

## 2. Methods

A thorough and detailed literature review was performed, searching the greatest reliable scientific databases, e.g., PubMed, Scopus, and Web of Science, utilizing effective, representative, and appropriate keywords like “insulin”, “glucose homeostasis”, “body weight”, “hypothalamus”, “appetite”, “hunger”, “satiety”, “orexigenic”, “anorexigenic”, “sugar substitutes”, “non-nutritive sweeteners”, “artificial sweeteners”, “aspartame”, “saccharin”, “stevia”, “acesulfame potassium”, “sucralose”, “ neotame”, etc. Only surveys written in the English language were included. In addition, clinical human surveys and in vitro and in vivo animal surveys were included. We did not include in the final analysis any gray literature, commentaries, editorials, letters to the editor, abstracts in congress proceedings, or any study published without a peer-reviewing process. Our investigation was extended by examining the reference lists of relevant studies and manually searching the reference lists of key journals, commentaries, editorial letters, and abstracts in congress proceedings. In addition, the recovered studies were carefully searched for relevant surveys mentioned in their manuscript. There was no time limitation concerning the included studies retrieved by the scientific databases.

## 3. Results

### 3.1. Hypothalamic Orexigenic/Anorexigenic Complex System: The Insulin Action

**Figure 2:**
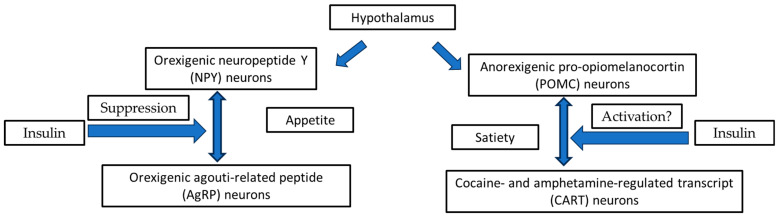
The hypothalamic orexigenic/anorexigenic complex system and the potential role of insulin.

As mentioned above, it is currently well-known that food intake is controlled by diverse, complex mechanisms, including nutrients, hormones, and neural signals. In this aspect, food intake appears to be controlled by the hypothalamus and hindbrain circuitries. Specifically, the hypothalamus arcuate nucleus constitutes the most important CNS region, participating in food consumption control [21]. Two crucial neuronal peptide cellular classes in the arcuate nucleus exist. These peptides can affect eating behavior: the orexigenic neuropeptide Y/agouti-related peptide (NPY/AgRP) neurons and the anorexigenic pro-opiomelanocortin (POMC) neurons [22]. Optogenetic or pharmacogenetic activation of NPY/AgRP neurons effectively promotes food consumption. In contrast, the optogenetic activation of POMC neurons suppresses eating and body weight increase [23]. Based on animal model studies of the hyperphagic syndrome and diabetes mellitus stimulated by streptozotocin, NPY/AgRP neurons were found to be more dynamic, and NPY and AgRP expression levels were enhanced, whereas POMC/CART neurons were shown to be to a lesser extent dynamic and POMC and CART expression levels were decreased [24]. Insulin and its activation signaling pathway seem to promote an anorexigenic effect in the hypothalamus by promoting a reduction in the levels of the neuronal peptides that stimulate appetite (NPY/AgRP), whereas it remains still questionable if insulin can activate satiety (POMC/CART) system [25]. The hypothalamic orexigenic/anorexigenic complex system and the potential role of insulin are illustrated in Figure 2.

**Figure 3:**
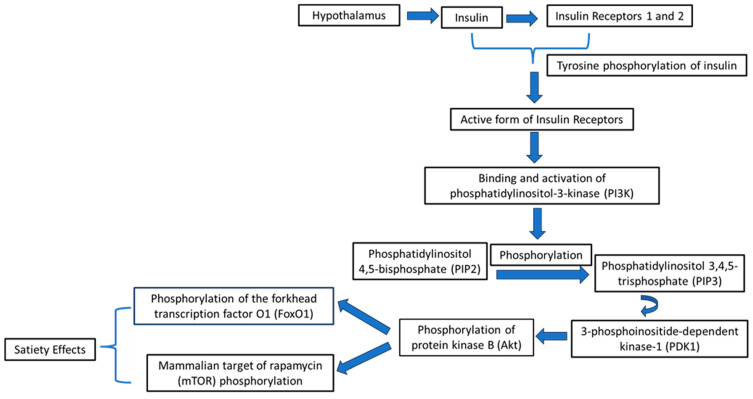
Potential molecular mechanisms of the anorexigenic action of insulin.

In the hypothalamic compartment, the insulin signaling begins by connecting to and stimulating the IR. The activated form of the IR then catalyzes the tyrosine phosphorylation of IR substrate 1 and 2 (IRS-1 and IRS-2) proteins. IRS phosphorylation can result in the connecting and stimulation of phosphatidylinositol-3-kinase (PI3K) [26]. Afterward, PI3K acts to phosphorylate the lipid phosphatidylinositol 4,5-bisphosphate (PIP2). The above event can then stimulate the release of phosphatidylinositol 3,4,5-trisphosphate (PIP3), which increases the levels of 3-phosphoinositide-dependent kinase-1 (PDK1) [27]. The active form of PDK1 then triggers the phosphorylation of protein kinase B (Akt) [28]. Afterward, the phosphorylated Akt enhances the levels of the mammalian target of rapamycin (mTOR) phosphorylation, resulting in food intake reduction [29]. The potential molecular mechanisms of the anorexigenic action of insulin are depicted in Figure 3.

Furthermore, the stimulated form of Akt transfers into the nucleus of the cells, inducing the phosphorylation of the forkhead transcription factor O1 (FoxO1), which omits the nucleus, resulting in satiety effects [30]. In fact, the FoxO1 transfer from the nucleus downregulates the transcription process of the orexigenic neuropeptides NPY and AgRP and upregulates the transcription process of the anorexigenic peptide POMC [30,31]. This increase in the expression levels of the POMC gene additionally stimulates the production of corticotrophin-releasing hormone (CRH) and thyrotropin-releasing hormone (TRH) by the paraventricular nucleus of the hypothalamus. Notably, both CRH and TRH have also been reported to act as anorexigenic neuropeptides [32,33].

### 3.2. The Cooperation of Insulin-Leptin Hormones in Hypothalamic Orexigenic/Anorexigenic Complex System

Over the last few decades, a significant research effort has been performed to identify the crucial signaling peptides in POMC and NPY/AgRP neuronal cells. One concurrent and conjoining site for both the IR and leptin receptor signaling is PI3K, and especially its subunit p110β [34,35]. Both the IR and leptin receptors act together to stimulate PI3K, regulating various functions, which can then enhance POMC neuronal excitability [34,35]. POMC neurons placed in the hypothalamus arcuate nucleus constitute crucial anorexigenic neurons. Both insulin and leptin depolarized POMC neurons by stimulating transient receptor potential (TRPC)5 channels [34,36]. Thus, insulin-and leptin-stimulated innervation of POMC neurons takes place through a receptor-facilitated stimulation of PI3K and the opening of TRPC5 channels that generate a vigorous inner cation flow, which depolarizes POMC neurons, enhancing their activation [34,36].

**Figure 4:**
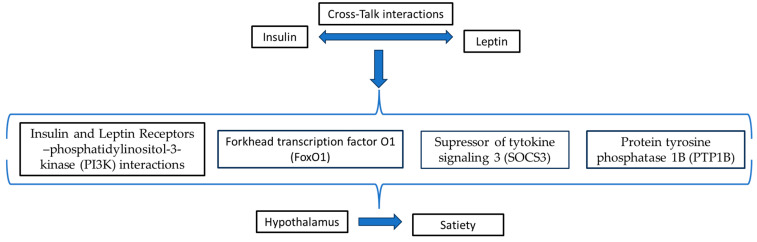
Potential crosstalk interactions between insulin and leptin that leads to satiety.

Recent studies have also indicated that insulin could enhance leptin-induced signaling. In this aspect, it has been well-established that leptin, secreted by adipose tissues in proportion to fat stores, can act in the hypothalamus to control eating behavior by rising satiety [37]. It has also been documented that both insulin and leptin may synergistically act to lower body weight and food consumption. Thus, the presence of a crosstalk between insulin and leptin could be considered as a crucial factor for the regulation of whole-body energy homeostasis [38]. Both insulin and leptin receptors use the same mechanism concerning their signaling pathways. Specifically, this common mechanism involves PI3K, while the IRS–PI3K interaction may be a potential mechanism by which the regulatory effects of both insulin and leptin on the reduction in food consumption are integrated [39]. Moreover, some evidence implies that FoxO1 seems to be considered as a mediator of the potential crosstalk between insulin and leptin, which is related to the regulation of food consumption [38,40]. In addition, the inhibitor of cytokine signaling 3 (SOCS3) and/or protein tyrosine phosphatase 1B (PTP1B) appear to share the suppressive effects on insulin and leptin signalings that are frequently observed in obesity [41]. The potential crosstalk interactions between insulin and leptin that lead to satiety are depicted in Figure 4.

### 3.3. Sucrose Substitutes (SSs) in the Control of Appetite/Hunger Complex System and Their Sweet Potency

Currently, several SSs are highly used as food additives [42,43]. SSs are also reported as artificial sweeteners, non-calorie sweeteners, non-nutritive sweeteners, and high-intensity sweeteners, which are very effective SSs, permitting the reduction in the energy content of foods and beverages, keeping elevated tastiness. Aspartame, saccharin, acesulfame, cyclamate, sucralose, neotame, and advantame are utilized worldwide as artificial sweeteners. Xylitol, sorbitol, erythritol, and other polyols, as well as steviol glycosides, belong to another category of sugar alcohols or naturally occurring sweeteners [42,43]. Currently, there are about 24 non-nutritive artificial sweetener surveys concerning their co-existence with the nature of our planet from approximately 38 different regions worldwide, spanning Europe, including the United Kingdom, Canada, the United States of America, and Asia [44]. Because of the distinct regulation of allowed artificial sweeteners in the United States and Europe, there are certain discrepancies and worries concerning the relationship between some sweeteners and their probable health dangers [45]. Compared to sucrose, all the above sweeteners are much sweeter. Hence, the use of diverse SSs may separately or concurrently decrease the levels of consumed sugar and thus decrease the calorie intake [46]. The impacts of SS sweeteners on the glucose metabolic process have extensively been examined [47,48]. Nevertheless, there are no conclusive data so far concerning the impacts of SS intake in comparison to either sugar, placebo, or nutritive low-calorie sweeteners intake on the potential clinical benefits or impairments regarding hemoglobin A1c (HbA1c), body weight, and side effects in individuals with diabetes mellitus type I or type II [47,48].

Furthermore, it is anticipated that food ingestion, including SSs, could not be an adequate and efficient technique for decreasing energy consumption and, subsequently, body weight decline [49]. Additionally, it is proposed that repeated consumption of SSs could even enhance the appetite for sweets or calories-dense foodstuffs [49]. The above consideration appears to have a considerable purpose in fighting obesity since an opposite association between body mass index (BMI) and sweet taste sensitivity was reported among overweight individuals [50,51,52]. For example, Proserpio et al. showed that people affected by obesity are characterized by decreased sensitivity to every basic taste compared to people with normal weight [53]. A poorer sweetness detection limit can probably result in higher sucrose- and carbohydrate-dense food intake to subsequently obtain the requested sweet-tasting strength. Nonetheless, some surveys do not verify the underlying relation between excess body weight and elevated desire for sweet foodstuffs [54,55]. On the other hand, the elevated consumption of non-caloric sweeteners has unexpectedly been associated with an elevated risk of cardiovascular diseases and depressive behaviors in adulthood, while it has also been related to an increased risk of obesity in children [56]. The consumption of SSs under specific conditions is thought to be harmless concerning the general population. However, there is only limited knowledge regarding their long-term health impacts [57]. Several review articles, as well as meta-analyses of clinical and experimental studies, were unsuccessful in establishing an agreement on this issue, supposing that SSs may exert favorable [58,59], detrimental [60,61], or trivial [62,63] impacts.

### 3.4. Experimental and Clinical Evidence and Potential Mechanisms for SSs in the Control of body Weight Gain 

There are currently several experimental studies evaluating the potential mechanisms of SSs in the control of body weight. More to the point, acesulfame (0.05%)-including water led to both body weight increase and fatty content increase in comparison with the 10% sugar-enriched water because of a better energetic efficacy in Sprague Dawley rats (5–6 weeks old male rats) [64]. Similarly, acesulfame (37.5 mg/kg body weight daily) resulted in a body weight increase with alterations in microbiome-produced metabolites in male CD-1 mice [65]. Notably, chronic acesulfame consumption inhibited sucrose taste sensitivity and decreased lingual sweet taste receptor expression, while hippocampus-dependent recall was also impaired [66]. In Sprague-Dawley rats (7-week-old male rats), the consumption of 0.05% aspartame considerably enhanced body weight as well as fatty mass, which could be ascribed to a raise in energy efficacy [67]. Moreover, aspartame consumption was related to both glucose intolerance and insulin resistance [67]. Using a rat model, including 29 male Wistar rats consumed everyday yogurt enriched with 20% sugar, 0.3% sodium saccharin, or 0.4% aspartame, supplemented with chow and water ad libitum, has been performed [68]. This study showed that a higher body weight increase was elevated by the usage of aspartame or saccharin, in comparison with sugar, and this weight increase was not related to calorie consumption [68]. Thus, it was speculated that a reduction in energy spending or elevation in fluid retention may be implicated [68]. 

Saccharin has been related to body weight increase, as administrating 0.3% saccharin or 0.4% aspartame for a period of one year resulted in an excessive body weight increase in adult Wistar rats [69]. The above body weight increase was not related to calorie consumption. The long-term saccharin consumption decreased the post-absorption energy expenditure at rest, and it was related to higher body weight increases relative to sugar in Wistar rats [70]. Accordingly, saccharin was shown to induce body weight increase without raising calorie consumption, which was not associated with insulin resistance in Wistar rats [69]. In addition, the impacts of long-term administration with drinks, including saccharin, on body weight and their potential mechanisms in post-weanling rats were examined [71]. In fact, saccharin solution drinking enhanced food consumption as well as energy consumption in male rats. In male rats, drinking saccharin solution enhanced TIR3 mRNA expression in the taste buds and ghrelin receptor mRNA expression both in the taste buds and hypothalamus; however, no impacts were observed in female rats [71]. Sucralose ingestion at 0.016% was shown to exert an analogous effect with that of aspartame to a smaller degree, though [67]. Moreover, sucralose elevated high-fat-diet (HFD)-stimulated liver steatosis. Additionally, sucralose administration raised reactive oxygen species (ROS) production and triggered endoplasmic reticulum stress in HepG2 cellular cultures [72]. In another study, wild-type and α-gustducin knockout (α-gust-/-) mice received a high-fat diet and force-feeding one time per day for a period of two months with water or equally sweet concentrations of sweeteners. This study showed that sucralose did not decrease body weight gain, fat pad mass, and insulin regardless of α-gustducin [73].

Van Opstal et al. [74] showed that the per os treatment with glucose resulted in immediate stimulation of the reward system. On the other hand, sucralose treatment led to only a slight, short-term response equal to that of water. On the contrary, another survey of the drinking of cocktails, including sucralose or allulose, resulted in a small reduction concerning the action of structures related to hedonic hunger in comparison with cocktails, including glucose or fructose [75]. Nonetheless, surveys exploring short-term impacts did not indicate any enhanced desire for sweetened foodstuffs next to repeated exposure to a sweetened-related stimulant [75].

Animal studies and human cross-over clinical studies in thin and obese persons did not find any considerable effect of SSs on incretin release [72,76,77,78,79,80,81,82]. In agreement with the absence of any impact on incretin release, there are also two additional cross-over clinical surveys, which did not find any impact on appetite from sucralose or aspartame-sweetened diet coke drinking in healthy and obese persons [77,78]. Also, randomized cross-over clinical studies documented lower reward and satisfaction signs from aspartame or sucralose intake in healthy people, thus indicating that calorie consumption is needed to induce a hypothalamus response [73,74]. Hence, it was anticipated that SSs could not trigger the food reward pathways in a similar manner to that of naturally occurring sweeteners like glucose [73,74].

Regarding certain kinds of SSs, glucose level control appears to not be affected by aspartame and steviol glycosides. No considerable impact on glucose concentrations and glycated hemoglobin (HbA1c) concentrations was observed next to acute or long-term aspartame consumption [76,77]. Regarding other SSs, glucose concentrations were not influenced by either acute saccharin intake in healthy persons and in those diagnosed with diabetes or acute acesulfame intake in healthy persons [76,83]. Also, glucose and HbA1c concentrations remained unaffected by acute or long-term sucralose intake in healthy individuals as well as in those diagnosed with diabetes [72,76,79,80,83,84]. Notably, short-term sucralose intake alone had not any impact on insulin sensitivity in healthy persons; however, sucralose-enriched beverages, including carbohydrates, or sucralose sachets used as supplements in carbohydrate-including beverages or meals, diminished insulin sensitivity in healthy persons [85,86,87].

An interesting meta-analysis exploring 9 longitudinal surveys did not find any substantial correlation between SS intake and body weight or fatty mass, but it found considerable relations with BMI [88]. Analogous moderate elevations in BMI were noted in another meta-analysis of thirty longitudinal surveys [89]. This survey documented that individuals with higher SS intake exhibited a higher risk of metabolic syndrome, obesity, cardiovascular events, and diabetes mellitus [89]. Recently, another meta-analysis reported similar findings [90]. The above surveys derived their data from 35 longitudinal surveys [90]. Moreover, data from diverse surveys, including an overall population of 17,934 people, demonstrated that greater dosages of SS were related to decreased body weigh increase; however, lower doses were related to lowered body weight increase. The above findings stayed similar in people in that both did and did not dynamically result in body weight decline [90].

Longitudinal clinical surveys that followed up large study populations for a prolonged interval showed harmful effects concerning body weight monitoring final outcomes [91]. Nonetheless, randomized, controlled surveys that had lower study populations but more appropriate controls mostly and frequently demonstrated either no effects or beneficial effects concerning SS consumption [91,92]. In agreement with the existing surveys on obesity, there was a stark polarization among longitudinal cohorts and randomized, controlled surveys on individuals with type 2 diabetes. Analogously, beneficial effects were noted in randomized, controlled surveys, whereas longitudinal cohorts showed harmful effects [93]. The justification for the above deviation among longitudinal cohorts and randomized, controlled surveys seems to mainly be ascribed to the controls [94]. Regarding longitudinal cohorts, the enrolled individuals lived freely, and further variables were included in the randomized, controlled surveys [94]. The above findings were compared using two very different strategies [93,94].

### 3.5. Do SSs Stimulate Insulin Secretion or Other Molecular Signs to Control Appetite/Hunger Complex System?

In conjunction with hunger/satiety homeostasis in brain areas, structures called hedonic brain areas constitute substantial modulators of feeding behavior. The above structure enhances the investigation and consumption of foodstuffs that lead to an intense sensation of pleasance [19]. The release of neuronal transmitters triggers hedonic paths through the hypothalamic compartment, including dopamine and endogenous opioid-related compounds, whose release rises afterward, receiving foodstuffs with high palatability [95,96]. De Araujo et al. supported evidence that the procedure of hedonic feeding includes a taste-nutrient (sensible or insensible) concept, and the insula was suggested as the joining structure concerning this sensible and insensible process [97]. After SS intake, it was assumed that merely in part, stimulation of food reward paths takes place, and the above activities have been attributed to the split of the effects of sweetening and energetic benefits [98,99]. A decline in reward response could raise the desire for feeding and, therefore, a food-searching attitude. Consecutive, no controlled intake of satisfactory sweetened foodstuffs could lead to a body weight increase [98,99].

It should be noted that SSs can stimulate insulin secretion at a lower level than natural sugars. More to the point, a randomized cross-over survey in healthy people showed that no cephalic insulin response took place during the tasting of aspartame, and an initial increase in insulin levels was noted during tasting glucose [100]. Likewise, no cephalic response to sucralose was observed in a randomized cross-over survey in healthy people [78]. Additionally, while naturally occurring sugars seem to activate the release of incretins and, in that way, induce β-cells to release insulin, artificial sweeteners cannot directly stimulate incretin release as the above seems nutrients-dependent [76,99,101]. Collectively, the existing clinical evidence implies that SSs cannot substantially influence insulin concentrations [102].

It has also been supported that SS intake may stimulate the release of incretin hormones by gut enteroendocrine cells in spite of their decreased or even the absence of energy content. Nevertheless, the above findings have exclusively been established by in vivo animal studies [103]. On the contrary, no incretin release was found in clinical studies stimulated by SSs [76,79]. However, it must be noted that the absence of incretin release has exclusively been observed so far in surveys in which SS consumption was not supplemented by foodstuffs consumption [102]. It has also been assumed that chronic exposure to SSs may exert a negative impact on cephalic phase insulin response [104]. Thus, eating the above kind of foodstuffs lacking energy content could lead to the absence of the cephalic phase insulin response, impairing postprandial glycemic control [104]. The available evidence so far does not fully verify that cephalic phase insulin response is an important crucial process controlling eating behavior in humans [105].

There are certain studies that supported the fact that SSs could improve insulin response next to oral glucose intake [106,107]. However, most of the surveys performed in healthy, normal-weight people did not find any impact of SSs, such as sucralose [79,108,109], aspartame [110,111], and saccharin [Horwitz 1988] on insulin secretion as a response to glucose. Thus, it was supported that the body weight of the enrolled individuals could affect hormone response [107].

Novel studies have documented that the neural reward mechanism is affected via the intestine, even if the precise processes through which this takes place have not been elucidated yet [112]. Noticeable enteroendocrine hormones (from proximal to distal gastrointestinal parts) with a well-recognized contribution in the control of foodstuffs consumption, as well as a possible impact in the modulation of neural structures of reward, have been reported. Moreover, certain gastrointestinal peptides located in the intestinal nervous system, rather than enteroendocrine cells, were found to be involved in the monitoring of eating behavior [112]. Several studies have suggested the potential impact of ghrelin on hunger and, consequently, mealtime beginning could expand to reward-motivated behavior/stimulus. Cholecystokinin and its receptors also exert an impact in regulating reward-associated behaviors [113]. Glucose-dependent insulinotropic polypeptide analogs can also reduce food intake and body weight in rodent models and clinical studies, both by themselves as well as in conjunction with additional intestinal peptide derivatives [114]. More to the point, glucagon-like peptide-1 analogs were found to decrease foodstuffs consumption, stimulation to intake foodstuffs rewards as well as conditioned reward responses when received peripherally, i.c.v. or through microinjection into reward-associated nervous areas [115]. Secretin was observed to stimulate brown adipose tissue as well as suppress appetite by suppression of orexigenic neuronal cells and activation of anorexigenic signals through POMC nerve cells [116]. Peptide tyrosine tyrosine can influence motivation to seek high-fat foods in a rodent model and modulate neural activity in reward-related brain regions in humans, suggesting that peptide tyrosine-tyrosine has some influence on the hedonic food intake [117]. Neurotensin, a 13 amino acid peptide, is expressed in the CNS and gastrointestinal tract. There is, notably, an i.c.v. infusion of neurotensin diminished feeding in starved as well as ad libitum-nourished rats, while a similar effect was noted with a peripheral therapeutic approach [118].

### 3.6. May SSs Increase the Risk of Adverse Effects?

**Figure 5:**
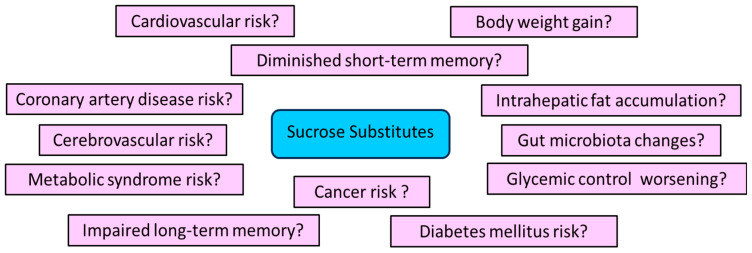
Potential adverse effects of sucrose substitutes.

Currently, several studies have shown that SSs may exert adverse effects on human health, even if the available data cannot be considered as conclusive (Figure 5). In this aspect, meta-analyses documented that SSs did not exert any impact on body weight or glycemic monitoring. Additionally, artificial sweeteners can modify the constituents of the microbiota and impair glycemic monitoring due to alterations in the intestinal microbiome. The immediate consumption of acesulfame also suppressed the taste response to sucrose. Moreover, a population-based prospective survey demonstrated that enhanced artificial sweetener consumption was related to all-cause mortality as well as to high risk of cardiovascular diseases, coronary artery disease, cerebrovascular disease, and cancer [119]. Another study has further reported that sweetened taste triggered higher stimulation of reward-associated nervous areas in self-mentioned diet soda drinkers than non-diet soda ones. In addition, the usual diet soda drinkers did not have any different nervous system response to nutritive and SSs [120]. The above findings have suggested that systematic SS intake could be related to alterations concerning the reward experienced from calories and non-calories sweeteners. Moreover, another study found that the incidence of SS intake was reversely related to the nervous system response to sucrose regarding the amygdala and insula [121].

The association of SSs with cardiovascular risk has not sufficiently been elucidated yet. In an initial survey of women, a greater consumption of artificially sweetened beverages was related to an enhanced probability of stroke, coronary heart disorder as well as all-cause mortality. Nevertheless, an enhanced consumption of SS beverages did not show any association with hemorrhagic stroke [122]. An important study has recently been published, which was performed on 103,388 individuals in the web-placed NutriNet cohort [123]. The above surveys explored aspartame, acesulfame, as well as sucralose; however, saccharin was not included in the analysis. SS consumption was related to an elevated likelihood of cardiovascular disease [123]. In accordance with a previous study, total artificial sweetener consumption was linked with an elevated probability of cerebrovascular disease. Acesulfame potassium and sucralose were also related to enhanced probability of coronary heart disease, whereas aspartame did not show any association with the likelihood of coronary heart disease [119]. Aspartate consumption also showed an association with an elevated probability of cerebrovascular disease, but acesulfame potassium and sucralose did not show any relation with the probability of cerebrovascular disorder. These results have suggested no benefits from using artificial sweeteners instead of sugar on cardiovascular disease outcomes [123]. In addition, an in vivo animal study, including 60 male Sprague-Dawley rats, also showed that all sweeteners tested (aspartame, sucralose, stevia, and xylitol) enhanced total cholesterol, low-density lipoprotein and triglyceride concentrations [124]. Short-term recall was considerably diminished in the aspartame group in the new object recognition task, and long-term recall was diminished in the stevia group [124]. The above metabolism and behavior findings supported evidence that the long-term consumption of SSs may be related to both peripheral and central adverse impacts [124].

Furthermore, the relationship of SS intake with cancer risk has recently been investigated. Specifically, 25 observational surveys (3,739,775 individuals) did not find any association of SS intake with cancer mortality or prevalence. On the other hand, European data demonstrated a correlation with cancer prevalence, in contrast to those of the United States or Oceania. Moreover, it was not found any correlation with cancer-related deaths in any of the involved areas. Nevertheless, a correlation with overall mortality was noted [125]. Altogether, these surveys did not observe any change in the effect of SSs on obesity-associated cancers or other cancers. Accordingly, the findings presented by the Melbourne Collaborative Cohort Study indicated that there was no association between SSs and obesity-related cancer risk [126]. Remarkably, aspartame constitutes one of the most examined food additives; however, aspartame safety is characterized by several discrepancies [127]. Formaldehyde, a metabolism by-product of aspartame, constitutes an established carcinogen, which is able to induce DNA damage, chromosome aberrations, and mitosis disturbances [128,129]. Another study documented that formaldehyde can exert a considerable effect on aspartame-stimulated carcinogenesis in the hepatic and lung tissues in mice [130,131]. However, it remains questionable as to what exact dose of aspartame may trigger carcinogenesis in humans, as the above evidence was derived from animal studies.

The meta-analysis of Yan et al. observed a definite impact of aspartame consumption on tumor malignancies [125]. Aspartame intake was found to enhance the prevalence of lymphoma and leukemia in rats through a dose-response way in an Italian survey [57,132]. Nevertheless, the European Food Safety Authority rejected this evidence because of the high frequency of infection and inflammation in the above animals, as well as the low certainty of diagnosis [57,132]. In the NutriNet-Sante population-based cohort survey, SSs (specifically aspartame and acesulfame) were related to a high likelihood of tumor malignancies [133]. Individuals who consume high amounts of artificial sweeteners exhibited a greater probability of overall cancer. Both aspartame and acesulfame were related to an enhanced likelihood of tumor malignancies. Elevated risks were also noted for breast carcinoma and obesity-associated tumor malignancies for all artificial sweeteners [133]. The above findings could be attributed to the interrelation between obesity and artificial sweeteners.

Another survey exploited Framingham Heart Study data to evaluate regular versus diet soft drink intake, assessing the probability of developing metabolic disorders [134]. The above study documented that the assigned individuals who received higher quantities of soda, either diet or regular, exhibited an enhanced probability of having symptoms related to metabolic syndrome. Accordingly, SS-enriched diet drinks were similarly linked with metabolic syndrome as regular drinks [134]. The above findings appeared similar to those observed in diverse relevant surveys [135]. The above survey has also suggested that SS consumption could not attenuate metabolic syndrome symptomatology but adequately exert analogous impacts of nutritive sweeteners [135].

Another longitudinal survey monitored its enrolled individuals from 2000 to 2007, recording both self-mentioned symptoms of diabetes and diet soda drinking [136]. This study recorded a 67% raised relative probability of diabetes type 2 concerning the participants receiving diet soda at least one time per day. The above findings were not dependent on baseline measures of adiposity [136]. A meta-analysis of longitudinal surveys assessing SSs documented an analogous elevated likelihood of developing diabetes concerning people who were classified in the highest quartile of SS intake compared with those classified in the lowest quartile [89]. In addition, the relative probability was observed to rise by 3% for every additional SS intake per day [89].

It should be emphasized that the assigned individuals concerning the currently available longitudinal surveys are at present living without any interventions, while they do not belong to a distinct sociodemographic population-based sample. Consequently, these data could be highly skewed due to unidentified co-variates. For instance, people who have specific consumer choices that involve diet beverages and other items enriched with SSs may probably present a trend to already be affected by obesity or to suffer from diabetes mellitus [91,137]. SSs are substantially more widespread among people affected by obesity in comparison with normal-weight or underweight individuals [138]. Hence, such surveys are reasonably restricted by inverse causality. Nonetheless, longitudinal studies have demonstrated that SSs could not really be capable of controlling or even decreasing body weight when the previously enrolled people are no longer being supported and advised by qualified personnel. The above assumption was verified through rodent experiments that revealed body weight increase, glucose intolerance, and/or inflammation related to SS intake [68,139]. Based on the above surveys, inverse causality may not be in danger since exogenous co-variates, like consumers’ choices, could be related to body perceptions that cannot occur in rodents.

Converting the above view, the findings of certain randomized, controlled clinical studies have demonstrated the probable beneficial effects of a strictly checked diet when combined with SS intake. The above surveys usually incorporate SSs in hypocaloric diets and control other co-variates [140]. The above findings showed whether SSs may be utilized as a component of more nutritional alterations if a person is cautiously adopting the completed dietary pattern. Thoroughly adopting the diet may also restrict calorie overcompensation. The above concern may be associated with the assumption that a person could think that as they lowered their calorie consumption with SSs, they would now be able to substitute those calories with other foodstuffs [141]. Consequently, they could recognize that they are consuming fewer calories than it does. The above concern could result in people receiving higher amounts of calories than they first consumed [142].

The fructose metabolic pathway could exert a detrimental impact on increasing energy overconsumption [143]. Additionally, receiving continuously high amounts of fructose related to a small energy turnover can lead to a long-lasting overproduction of intrahepatic trioses-phosphate production that can secondarily result in the development of liver insulin resistance, intrahepatic fat accumulation, and elevated blood triglyceride concentrations. In the long term, the above impacts could promote the development of metabolic and cardiovascular disorders [143,144].

A number of randomized controlled clinical studies evaluated the short-term (from a few days to a few weeks) metabolic impacts of high-fructose-containing caloric sweeteners (FCCSs) diets in healthy, normal-weight, and overweight individuals. However, the general interpretation and clinical significance of these surveys vary broadly, and their evidence showed considerable enhance of both fasting and postprandial blood lipid levels in the enrolled individuals receiving high-FCCS diets [145]. The above raise seems to be strongly significant but quantitatively modest, and absolute blood triglyceride levels remain in the normal range concerning most normal-weight individuals, even in individuals receiving very elevated dietetic fructose amounts [146]. However, it should not be disregarded the probability that blood triglycerides’ may be increased to levels related to a higher likelihood of developing cardiovascular diseases in overweight and insulin-resistant individuals [147]. Notably, a few clinical studies found that individuals who received a high-energy, high-fructose diet can significantly collect higher amounts of fat in visceral adipose depots than control individuals who adopted isocaloric high-glucose dietary patterns [148,149].

Additionally, only 25% of parents may well recognize foodstuffs and beverages that were enriched with SSs, revealing that several adults and children may receive SSs by mistake [150]. The growing intake of SSs amongst children may be a result of marketing promotion strategies, which present SSs as a healthy alternate to sucrose in an attempt to limit the child obesity epidemic [150,151], which seems to be a worldwide phenomenon. Forshee et al. [152] evaluated the beverage intake of American children (n = 331, age 6–19 years old) and recorded that elevated artificially sweetened beverages consumption was considerably related to higher BMI, which was in agreement with a similar survey (n = 385, American children at the age of 11–13 years old) by Giammattei et al. [153]. Accordingly, Laverty et al. explored the nutritional habits of 13,170 children in the United Kingdom and observed that everyday artificially sweetened beverage intake was associated with an elevated body fat content and BMI at the age of 11 years old [154]. In the International Study of Childhood Obesity, Lifestyle, and the Environment, including 6162 children from 12 countries covering a range of financial and human development, Katzmarzyk et al. recorded a gender-specific and dosage-dependent positive relation between diet soda drinking and BMI z-score, body fat proportion, and risk of obesity in girls [155].

Recently, recovering data from the 2009–2014 US National Health and Nutrition Examination Survey (NHANES), Sylvetsky et al. documented positive relationships between SS intake and obesity [156]. In adolescence (ages 12–19 years), odds of obesity were consistently higher in SS consumers than non-consumers, while in children (aged 2–11 years), the above relationship was directly noted in boys and adolescences who were Hispanic [156]. Insulin and glucagon can stimulate signaling pathways in the nervous system to modulate energy and glucose balance [157]. In contrast to the periphery, insulin and glucagon signaling in the CNS cannot exert opposed metabolic impacts, as both of them can suppress food consumption and body weight increase [Filippi 2018]. They send their signals by divergent pathways and change dissimilar neuronal populations, supporting evidence for a corresponding action of the two hormones in modulating eating behavior. In accordance with its systemic impact, insulin signaling in the CNS reduces glucose production [157]. The above studies support sufficient evidence that glucose and fructose exert very different actions. More to the point, it is implied that glucose immediately provides energy, and fructose contributes to the storing of energy [158]. The action of fructose is firstly time-restricted; however, over a lifetime, the frequent mitochondrial oxidative stress leads to mitochondrial division and drop-out, resulting in more long-lasting alterations, which block one into an obese status and promote the aging procedure [159].

Concerning natural SSs, steviol is consumed worldwide as a sweetener; however, its usage must be careful. A recent in vitro study showed that steviol can exert cytotoxic, genotoxic, and mutagenic actions in the levels and circumstances used in human lymphocyte cellular cultures [160]. Specifically, steviol reduced the amount of lymphocytes because of the decline of CD4+, CD8+, and CD4+CD8+ sub-populations and increased the extent of DNA damaging, leading to a gradually increasing frequency of structural alterations in the lymphocyte chromosomal sets [160]. In contrast, no substantial change in the production of chromosomal abnormalities and micronuclei was found among the groups receiving stevia and the negative control at 24 and 48 h treatment intervals in human peripheral blood lymphocytes [161]. On the other hand, a case-report on a 54-year-old man with obstructive sleep apnea suffered from restless legs syndrome symptoms when he received a stevia extract-derived no calories sweetener [162]. Moreover, steviol glycosides include a steroidal structure and thus could act as endocrine disruptors in the human body [163]. Reporter gene assays (RGAs), H295R steroidogenesis assay, and Ca^2+^ fluorimetry-based analyses utilizing human sperm cells were applied to assess the endocrine disturbing potency of two steviol glycosides: stevioside and rebaudioside A and their metabolite steviol. This study provided evidence for the potency of steviol to function as a possible endocrine disruptor [163]. However, most of the studies have suggested that stevia may not stimulate teratogenicity, mutagenicity, or carcinogenesis and could only lead to no acute and subacute toxicity, verifying its safe use [164].

## 4. Discussion

For millions of years, the basic function of insulin is its secretion by β-cells of the pancreas as a physiological response to carbohydrate intake in order to transfer the produced by peptic ingestion glucose into the adipocytes, hepatocytes, and muscle cells [24,165]. A less familiar insulin action is its anorexigenic effect by its transfer into the hypothalamus of the brain, increasing the long-term hedonic properties of nutrients, which can control food intake and thus decrease excessive calories and control body weight maintenance [24,165]. In contrast, SS intake does not influence the appetite/hunger complex system of the hypothalamus, and subsequently, SSs cannot satisfy the hedonic aspect of food and beverage intake [19]. SS supplements in diets do not have any positive effect on body weight decline or lowered weight increase without energy limitation. There are prolonged and novel worries that the introduction of SSs in any dietary pattern raises energy consumption and contributes to the development of obesity [59]. Thus, they cannot activate the anorexigenic neuropeptides of the hypothalamus, enhancing the sense of hunger and thus the intake of excessive calories, which may lead to body weight increase if a well-balanced diet is not carefully applied at a daily frequency.

Even if increased SS consumption has historically been associated with the increasing prevalence of obesity, the existing results remain no conclusive and are not convincing yet [98]. There is substantial evidence that either the addition of saccharin or aspartame is related to an enhanced sense of hunger [75]. Wistar rats treated with yogurt enriched with either saccharin or aspartame and a free chow diet for 3 months resulted in elevated body weight increase than animals treated with the similar free chow diet plus yogurt enriched with sugar [68]. Saccharin and aspartame stimulated rather fewer calories from yogurt consumption in comparison with sugar. However, increases in calories from chow consumption efficiently compensated for declines in calories from yogurt in such a manner that there was a comparable overall calorie consumption amongst all groups next to the 3-month duration of the experimentation [68]. Male Wistar rats, which received *Stevia rebaudiana* dry leaves infusion at a concentration of 0.94% concurrently with the diet, did not show any considerable alterations compared to the other groups in body mass (326–401 g/male rat) [166]. The intake of a per-day infusion of *Stevia rebaudiana* dry leaves did not exert any beneficial or harmful impact on the decrease in body mass [166]. Hence, it is highly recommended that long-term prospective surveys be accomplished in order for more reliable and accurate conclusions to be drawn.

A thought concerning SSs is that they cannot promote satiety and that they could exert an opposed impact by enhancing hunger, resulting in direct or delayed energy benefits. Most findings derived from the small, short-term surveys are contradictory concerning the appetite impact of SSs. In this aspect, Mattes and Popkin supported from these surveys that SSs instead of sugar-enriched beverages may result in incomplete energy compensation [59]. The above short-term data seem to be attractive; however, long-lasting surveys are recommended to demonstrate the impact of chronic intake of SSs and the precise role that they could exert in satiety and metabolism. At the same time, a gradually increasing number of studies support evidence for a positive relationship between SS intake and body weight increase, metabolic syndrome, and type 2 diabetes. Hence, it is estimated that SSs may not be such safe molecules since they are probably able to influence diverse neurohormonal and biochemical pathways related to energy modulation and glucose level control [167]. More to the point, frequent intake of SSs can promote the conditioned orosensory stimulus of sweet taste in the absence of post-ingestive nutritive outcomes, which could lead to a suppression of conditioned cephalic phase responses and postprandial glycemic regulation [167]. The above inhibitory effect could carry on even if the sweet taste is once again supplemented by caloric content because of a reduction in the conditioned stimulus. This conditioned stimulus-unconditioned stimulus uncoupling may weaken the capacity of sweet taste to predict energy availability and properly direct consumption. Thus, SS intake could diminish energy homeostasis by affecting the predictive interrelationship between sweet taste and post-ingestive effects [167]. Characteristically, there are currently some data supporting evidence that both aspartame and sucralose cannot affect cephalic insulin phase response and postprandial glycemic regulation [78]. It has also been supported that chronic exposure to SSs could be related to a harmful effect on cephalic phase insulin response [104]. This means that consuming this kind of food lacking energy load could weaken or even minimize the cephalic phase insulin response, diminishing postprandial glycemic control. Nevertheless, there are still few data so far concerning the mechanism governing the cephalic phase responses to SS intake. Recently, it should also be noted that Langhans et al. have supported an urgent attention on the impact of brain receptors in the whole insulin response to eating rather than being dependent solely on the classical cephalic phase insulin response definition [168].

Low-calorie sweeteners (LCSs) are also frequently utilized as SSs [169,170]. Several recognized media websites emphasize warnings contrary to the consumption of such sweeteners due to their probable side effects, like inflammation. Nevertheless, there are quite restricted and accurate data so far concerning safety issues. A PubMed research of articles written from 2010 to 2020 was performed by substantial review studies, leading to 833 articles, of which 12 appropriate surveys were retrieved [98,170]. Acute side effects related to the intake of SSs and LCSs were quite scarce. A low number of surveys have reported acute side effects, such as moderate gastrointestinal disruptions, headaches, lightheadedness, hypersensitivity reactions, diminished spatial orientation, depressive symptoms, and pain [171]. Moreover, minor research has been performed since 2010 to establish the above caution declarations to consumers concerning the potential acute side effects of SSs and LCSs [170].

LCSs can offer sweetness with modest or no calories. Nonetheless, every LCS’s exclusive chemical structure exhibits the potency to prompt distinct sensory, physiological, and behavioral responses, which are able to influence body weight. In a relevant survey, 154 individuals were randomly enrolled to intake 1.25–1.75 L of beverage enriched with sugar (n = 39), aspartame (n = 30), saccharin (n = 29), sucralose (n = 28), or rebaudioside A (rebA) (n = 28) per day for 3 months [172]. Sugar and saccharin intake considerably elevated body weight compared with aspartame, rebA, and sucralose, whereas body weight alteration was directionally negative and smaller for sucralose in comparison with saccharin, aspartame, and rebA consumption. In view of the above findings, LCSs must be considered as different entities due to their different impacts on body weight [172].

Additionally, in obesity and diabetes, insulin action as an anorexigenic hormone in the hypothalamus is suppressed through insulin resistance [24]. Hence, hyperphagia occurs, which further provokes hyperglycemia as well as insulin resistance, resulting in a malicious circle in which the patient is not able to control their requirement to consume. No controlled calorie consumption stimulates a boost to reactive oxygen species, affecting the anti-oxidant defensive systems of the cells (oxidative stress). Reactive oxygen species trigger stress-related kinases, like c-Jun N-terminal kinase as well as p38 mitogen-activated protein kinase, which can stimulate the phosphorylation of serine residues in IRs, that suppresses the insulin signaling pathway, retaining ongoing the system of insulin resistance [24].

Notably, maternal SS consumption throughout gestation has been related to elevated body weight increase in newborns, according to data derived from clinical surveys [173]. A significantly increased weighted mean difference in BMI z-score was noted in newborns in the first year of their life with SS consumption throughout gestation in comparison with control. A substantial dosage-response relation of SS consumption with an elevated weighted mean difference was also observed in newborns in the first year of their life [173]. In the CHILD birth cohort (n = 2298), mothers’ SS beverage consumption throughout gestation and child body components aged three years were determined after adjustment for mothers’ BMI and other possible confounding factors. To explore causality mechanisms, pregnant C57BL6J mice received SSs at dosages related to human eating (42 mg/kg/day aspartame or 6.3 mg/kg/day sucralose) and examined newborns until three months of age for body weight, adiposity, adipose tissue morphology and gene expression, glucose, and insulin tolerance [89]. The above survey, by triangulating data from humans, mice, and cultured adipocytes, provided novel evidence that mothers’ SS intake throughout gestation could enhance the probability of developing obesity in newborns by exerting considerable impacts on adiposity and adipocyte differentiation [89]. At present, the impacts of eight extensively consumed SSs on fat accumulation were determined in Caenorhabditis elegans. Possible mechanisms concerning eating and locomotion behaviors, lipid metabolism, and neural control were investigated. The above survey found that acesulfame, aspartame, saccharin, sucralose, and cyclamate promoted lipid accumulation at μg/L concentrations, revealing obesogenic potency. Behavioral research indicated that acesulfame, aspartame, saccharin, sucralose, and cyclamate predisposed more consumption in the energy consumption aspect compared to the locomotion in the energy intake one [174].

Most of the clinical surveys performed did not find any substantial or beneficial impacts of SSs on body weight and glycemic control. However, it must be mentioned that the nutritional intervention period of most of the existing surveys was quite short. Hence, additional well-designed, long-lasting randomized human surveys exploring the impacts of different artificial sweeteners and their potential effects on intestinal microbiome, body weight, and glucose level control, as well as the implicated mechanisms, are highly recommended [102]. Notably, the gut microbiota has been related to diverse physiological functions, while emergent evidence connects it with obesity and other metabolic dysfunctions [175]. The diet can exert a crucial impact, affecting the intestinal microbiome components, either positively or harmfully, by modifying certain bacterial species and modifying the metabolites created within the intestinal environment [176]. In this aspect, several studies have explored the potential associations between SS intake and alterations in microbiomes or bacteria, raising the possibility that SSs may exert impacts on human health through interactions with gut microbiome [177]. A dysbiotic impact of SSs was noted in certain clinical studies; however, several other randomized controlled clinical studies revealed an absence of substantial effects on intestinal microbiome components [176,177]. The above discrepancies may be ascribed to the fact that most currently available clinical surveys presented several differences concerning the study sample size of the enrolled people, their eating behaviors, lifestyle, age, as well as the presence of comorbidities [176,177].

Moreover, it should be noted that the majority of the reviewed studies presented several limitations. There are several conflicting results that may be ascribed to the different study and methodology designs, the different dosages and duration of SS treatment, as well as the different types of animal models or the different human population characteristics. Concerning the human clinical studies, the age and the gender of the enrolled individuals, as well as the presence of any pathological state such as obesity, diabetes mellitus, or metabolic syndrome, could be responsible for the contradictory results. The genetic profile of each study population, in conjunction with the nutritional and lifestyle habits of each study population, could act as confounding factors that may exert a significant impact on the derived results. As a representative example, certain clinical studies had a retrospective design, whereas others had a prospective design. Moreover, some of the prospective cohort studies showed different results from the randomized controlled clinical trials, while the presence or absence of a placebo group may also be a potential factor for the existing conflicting results. It should also be noted that certain animal and human studies used more than one SS, whereas others utilized only one specific SS. Moreover, there is a trend to pay more attention to the evidence from observational studies compared to randomized controlled clinical trials. However, randomized controlled clinical trials should require a greater priority in the established hierarchy of evidence as they can better infer causality than observational cohort studies [48]. All the above potential differences and limitations of the existing literature constitute a crucial challenge in interpreting conflicting results, highlighting the strong demand for additional research in order for a more precise conclusion to be drawn.

The acceptable daily intake (ADI), mg/Kg body intake) and the amount by which SS is sweeter than sucrose constitutes additional concerns that should be taken into consideration when designing and performing future studies [178]. ADIs are usually established by those recommended by the Joint Expert Committee on Food Additives (JECFA), apart from European surveys in which ADIs are mostly recommended by the European Food Safety Authority (EFSA) [178]. SSs are becoming increasingly available on the market during the last few years, and regardless of their comprehensive assessment concerning their safety before their authorization, serious arguments remain about the effects of their intake on human health [179]. Although SSs are strongly sweet, they are structurally diverse, and a restriction of several surveys exploring the health effects of intake is that they frequently fail to discern consumptions of distinct SS [179].

There are also several studies that have reported gender variations concerning the death rates related to diabetes. This is an important issue concerning SS consumers with diabetes. In this aspect, certain meta-analyses, via internal, within-survey comparisons between female and male assigned individuals, have demonstrated that women with diabetes exhibit a considerably greater probability of developing coronary heart disease, stroke, and gastric carcinoma in comparison with the affected men [180]. More to the point, a meta-analysis conducted on 64 cohort studies showed that the elevated likelihood of stroke relating to diabetes was considerably enhanced in women compared to men, independently of gender differentiations in several important cardiovascular risk factors [181]. Another meta-analysis by the same research group was performed on the same cohort studies demonstrated that women with diabetes exhibit more than a 40% greater probability of incident coronary heart disorder compared to men with diabetes [182]. Accordingly, a meta-analysis conducted on 38 cohort studies found that women diagnosed with diabetes had a considerably higher risk of gastric cancer compared to men with diabetes [183].

Furthermore, it should be emphasized that international authorities consider SSs, and especially artificial sweeteners, as safe as they do not pose any health-related problems when consumed within their recommended ADI [102,119]. However, no definite safety claims have been made concerning their impacts on non-communicable diseases, like obesity and diabetes mellitus [102,119]. This concern could be ascribed to the fact that artificial sweeteners exhibit distinct chemical structures and are metabolized differently, as some but not all are digested or fermented [102], 119]. In this aspect, several systematic reviews and meta-analyses based on longitudinal clinical surveys in healthy individuals showed a positive association between artificial sweetener intake and the prevalence of diabetes mellitus type II, independently of adiposity [57]. However, long-term animal and human studies exploring the underlying molecular mechanisms and the potential effective duration treatment of artificial sweeteners remain scarce and, therefore, warranted. A cross-sectional study conducted on patients diagnosed with diabetes mellitus type II showed that artificial sweeteners increased insulin resistance and that the duration of usage of artificial sweeteners exhibited a direct impact on insulin resistance [184]. However, the currently available animal and human studies used diverse SS dosages and treatment durations, highlighting the robust request to accomplish upcoming surveys exploring whether the duration of using SSs, such as artificial sweeteners, may affect insulin resistance.

Lastly, it should be highlighted that the old perception that a patient with diabetes mellitus type 2 must strictly avoid as much possible glucose uptake has collapsed, as most of the common foods include carbohydrates. Thus, it is reasonable that a patient with type 2 diabetes is unavoidably exposed to glucose by the diet. So, a personalized approach should be developed for each diabetic patient separately according to their specific characteristics. Thus, it could be assumed that the β-cells of the pancreas should be exposed even to low levels of glucose in order to not remain completely inactive to produce even low levels of insulin. In this case, the β-cells exposed to small amounts of glucose may receive stimulation signs to try to function by producing some amounts of insulin, depending on the specific characteristics of each diabetic patient. In contrast, a diabetic patient who frequently consumes SSs does not boost their β-cells to produce insulin, which may lead them to their full inactivation.

## 5. Conclusions

The impact of SSs on body weight gain is multidimensional, depending on diverse factors like dosage, the chemical structure of the compound, and host genetics. As a result, the currently available studies cannot provide conclusive evidence. There is currently substantial evidence that supports the fact that SSs could stimulate pro-inflammatory alterations in the intestinal microbiome and intestinal wall immunological reactivity, which may harmfully influence people with or vulnerable to chronic inflammatory environments. The above concerns are of great importance for individuals diagnosed with chronic digestive inflammatory diseases. Taking into consideration that there are differentiations in how people process nutrition signals, only a small number of surveys focus on gender or disorder predisposition differences and how they can influence the outcomes when SSs are consumed. Surveys that controlled these co-variates indicated impaired outcomes when SS products are consumed, aiming at preventing adiposity-associated disorders like hypertension, dyslipidemia, and diabetes.

Changes in the intestinal microbiota regarding a more inflammatory pattern of intestinal microbiome constitute a disrupting finding in acute as well as long-lasting consumers of SSs despite the baseline weight or disorder. Notably, there were several surveys that reported long-lasting damage to the neurohormonal control of satiety in chronic consumers of SSs. Future research on SSs must take into consideration specific differences, such as the gut microbiota, the kind of SS, and the period of former SS administration. Additionally, more long-term prospective cohort studies, as well as well-designed randomized, controlled clinical trials, are highly recommended to effectively indicate the impact of SSs on human health. Future clinical studies should be performed separately for each SS, as each of them has a different chemical structure, which may affect differentially their molecular mechanisms of action as well. Moreover, future clinical studies should be mostly directed at study populations with similar characteristics such as age, gender, BMI, and the presence of specific diseases (e.g., diabetes mellitus, metabolic syndrome, cardiovascular diseases, cancer, neurodegenerative disorders, etc.). Future research should also be focused on the potential long-term effects of SSs on gut microbiome and inflammatory-related conditions. Future research directions are also recommended to focus on the study and methodology designs, the exact target populations, the dose and treatment duration, and the investigation of each specific SS, also giving an emphasis on exploring individual differences and examining long-term effects on gut microbiome and inflammation.

## Data Availability

The data of the present study are available from the corresponding author for private use only.
